# Effects of Nanoparticle-Based Activating Flux with Sodium-Silicate Solvent on Activated Gas Tungsten Arc Welded Inconel 718

**DOI:** 10.3390/ma19132776

**Published:** 2026-06-30

**Authors:** Sebastian Balos, Nemanja Kljestan, Miroslav Dramicanin, Petar Janjatovic, Marko Knezevic

**Affiliations:** 1 Department of Production Engineering, Faculty of Technical Sciences, University of Novi Sad, Trg Dositeja Obradovića 6, 21000 Novi Sad, Serbia; sebab@uns.ac.rs (S.B.); dramicanin@uns.ac.rs (M.D.); 2 Department of Mechanical Engineering, University of New Hampshire, Durham, NH 03824, USA

**Keywords:** ATIG welding, sodium silicate-based flux, SiO_2_ and TiO_2_ nanoparticles, nickel alloy, molten metal flow

## Abstract

Activated Tungsten Inert Gas (ATIG) welding employs an activating flux to increase penetration and improve productivity compared with the conventional Tungsten Inert Gas (TIG) process. Conventional fluxes typically consist of metallic oxides dispersed in alcohol- or acetone-based solvents. In this study, a novel flux composed of SiO_2_ and TiO_2_ nanoparticles suspended in a sodium-silicate solvent was used for welding Inconel 718. The proposed flux achieved full penetration of a 7 mm thick plate at 160 A DCEN using 60° and 90° electrode tip angles, without visible distortion or defects in the examined cross-sections. Microstructural characterization revealed notable changes in the content, morphology, and size of Nb-rich interdendritic constituents consistent with Laves phase formation compared with welds produced without flux. ATIG specimens contained a lower amount of these brittle intermetallic constituents, which exhibited a less branched and more coagulated morphology despite the lower cooling rate. As a result, a greater fraction of alloying elements remained available for dendrite reinforcement rather than being segregated into Nb-rich interdendritic regions, leading to higher weld-metal microhardness in the ATIG60 specimen than in TIG welds. These observations were attributed to enhanced weld-pool stirring caused by molten metal flow toward the weld center and downward through the weld pool, consistent with the reversal of Marangoni convection.

## 1. Introduction

Gas Tungsten Arc Welding (GTAW), also known as Tungsten Inert Gas (TIG) welding, is a widely used fusion welding process recognized for producing high-quality welds with excellent control over weld geometry and metallurgical properties [1,2]. Owing to its versatility, GTAW can be integrated with welding tractors, orbital welding systems, robotic platforms, and Wire Arc Additive Manufacturing (WAAM) processes [3]. The process employs a non-consumable tungsten electrode to generate an electric arc, while an inert shielding gas protects the weld pool from atmospheric contamination [4,5]. Despite these advantages, several limitations restrict the productivity of GTAW, including relatively low deposition rates, shallow penetration, low travel speeds, and difficulties in welding thicker sections in a single pass [6].

Single-pass penetration in conventional GTAW is typically limited to only a few millimeters, which often necessitates edge preparation and multiple welding passes [4,5,6,7]. To overcome these limitations, several process modifications have been developed, including Activated Tungsten Inert Gas (ATIG) welding. The ATIG process was first introduced in the 1960s at the Paton Welding Institute by Gurevich, Zamkov, and Kushnirenko for welding titanium alloys using oxide-based activating fluxes dispersed in volatile solvents [8]. Since then, the process has attracted considerable attention because it can significantly increase penetration depth while maintaining the inherent advantages of conventional GTAW [9].

An activating flux typically consists of metallic oxide particles dispersed in a solvent carrier. The metallic oxides may include TiO_2_, SiO_2_, Cr_2_O_3_, NiO, Fe_2_O_3_, Mn_2_O_3_, Co_3_O_4_, and MoO_3_, while commonly used solvents include acetone, methanol, ethanol, and methyl ethyl ketone [10]. The flux is applied as a thin coating to the weld surface prior to welding. Numerous studies have demonstrated that ATIG welding can substantially increase penetration in a wide range of materials, including stainless steels, titanium alloys, nickel-based superalloys, and structural steels [11].

Several mechanisms have been proposed to explain the penetration enhancement observed in ATIG welding. The most widely accepted explanation is the reversal of Marangoni convection. In conventional TIG welding, molten metal flows outward from the weld center toward the pool edges, producing a relatively wide and shallow weld pool. Oxygen originating from the activating flux alters the surface-tension gradient at the weld-pool surface, reversing the direction of fluid flow toward the weld center and downward into the joint [12,13]. This inward flow promotes deeper penetration and a narrower weld profile. Arc constriction has also been identified as a contributing mechanism, increasing current density and concentrating heat within the weld pool [14,15]. Although the relative importance of these mechanisms remains a topic of discussion, both are widely recognized as contributors to the enhanced penetration achieved in ATIG welding.

In recent years, considerable attention has been devoted to the development of novel activating flux formulations. Numerous patents and scientific studies have reported fluxes based on different combinations of metallic oxides and nanoparticles, including TiO_2_, SiO_2_, Cr_2_O_3_, and rare-earth oxides [16]. Nanoparticle-containing fluxes have shown particular promise because of their high surface area and enhanced interaction with the welding arc and molten weld pool [17,18,19]. Several studies have reported significant increases in penetration and modifications of weld-metal microstructure through the use of nanoparticle-based activating fluxes [20,21,22,23].

Despite the demonstrated effectiveness of conventional ATIG fluxes, most formulations rely on highly volatile solvents such as acetone, methanol, ethanol, and methyl ethyl ketone. Evaporation during storage and handling can alter flux composition and reduce process consistency. Therefore, the development of alternative solvent systems with improved stability and shelf life represents an important opportunity for further advancement of the ATIG process. Sodium silicate is widely used as a binder in shielded metal arc welding (SMAW) electrode coatings, where its primary function is to provide coating integrity and process stability. Its potential application as a solvent and carrier medium for ATIG fluxes, however, has received very limited attention.

Accordingly, the objective of the present study was to investigate a novel ATIG flux consisting of SiO_2_ and TiO_2_ nanoparticles dispersed in a sodium-silicate solvent for welding Inconel 718. Inconel 718 was selected because of its widespread use in aerospace and energy applications and its sensitivity to welding-induced microstructural changes, particularly Nb segregation and Laves phase formation. These characteristics make the alloy a suitable candidate for evaluating the influence of novel ATIG flux formulations on both weld penetration and weld-metal microstructure. The proposed flux was evaluated with respect to weld penetration, weld geometry, microstructure, phase morphology, and hardness. Particular attention was devoted to understanding the influence of the sodium-silicate-based flux on weld-pool behavior and microstructural evolution. The work builds upon the concept disclosed in US Provisional Patent Application # 63/362625 [24].

## 2. Experimental Part

### 2.1. Materials and Welding Procedure

Within the framework of this study, the ATIG welding process was done on IN-718 base metal (BM), with the chemical composition presented in Table 1. The plates used for welding were 7 mm thick, waterjet cut to dimensions of 100 mm × 50 mm. All welding trials were conducted using specimens of identical dimensions to ensure consistent thermal boundary conditions and enable a direct comparison of the investigated activating fluxes. Welding was done on specimens with I-preparation (square butt preparation) with no gap, without the flux and with the flux described in US Provisional Patent Application # 63/362625 [24].

The activated flux consisted of the active substance and the solvent in the form of sodium-silicate (sodium-metasilicate; Na_2_SiO_3_), a new and novel type of solvent, to date only used for ATIG welding of AISI 304L austenitic stainless steel, as described within the framework of US Provisional Patent Application # 63/362625 [24]. Sodium-silicate is not readily evaporable, as are acetone and alcohol (ethanol or methanol), and thus possesses a major advantage in having a relatively long shelf life. A total of 7 wt.% of the flux was the mixture of hydrophilic nanoparticles: 20 wt.% of 20 nm TiO_2_ and 80 wt.% of 40 nm SiO_2_. The flux was fabricated by weighing the components using a Type 2615 (Tehtnica, Zelezniki, Slovenia) analytic balance. Afterwards, the components were mixed with the solvent using a MM530 (Tehtnica, Zelezniki, Slovenia) magnetic stirrer for 10 min. 

Prior to welding, all specimens were cleaned and stored under laboratory ambient conditions (20 °C). No preheating was applied. The plates were aligned and joined at the edges using small tack TIG welds to ensure proper fit-up and prevent relative movement during welding. Ceramic backing segments were used along the root side to provide support for weld pool formation and to ensure consistent root geometry. Due to their low thermal conductivity, the ceramic backing served primarily as a geometrical support rather than a significant heat sink. All experiments were performed using identical fixturing, tack-welded assembly conditions, and backing arrangements to ensure repeatable boundary conditions across all welding trials.

Welding was performed on a HandyTIG (Lorch Schweißtechnik, Auenwald, Germany) 200 AC/DC device, with 160 A DCEN (Direct current with a negatively charged electrode) and 12.7 mm diameter nozzle. Argon shielding gas was used, with a flow rate of 10 L/min. An electric arc was established by a 2% thoriated tungsten electrode (red-marked) with a diameter of 2.4 mm, with a 6 mm protrusion of the electrode from the nozzle, which was 2 mm from the BM. Sharpening of the electrode was done by using the TEG 4.0 grinder device (Lorch Schweißtechnik, Auenwald, Germany). A welding torch was attached to the Trac WL Batt welding tractor (Lorch Schweißtechnik, Auenwald, Germany), which enabled a stable 40 mm/min welding speed and fixed vertical (90° from horizontal) position of the torch. No weaving and consumables were used. Before welding, surfaces were cleaned with acetone, 4.8 g of the flux was applied by a brush (10 mm × 2 mm size) and spread over the whole length of the specimens, 20 mm wide (10 mm over each base metal). After the application, CO_2_ was applied for 5 s to dry the specimens, with subsequent welding. Five specimens were welded: control specimen welded with the electrode sharpened to 60° tip angle and without the flux (designated as TIG60); specimen welded with the electrode sharpened to 30° with the flux (ATIG30); specimen welded with the electrode sharpened to 60° with the flux (ATIG60); specimen welded with the electrode sharpened to 90° with the flux (ATIG90); specimen welded with the electrode sharpened to 180° with the flux (ATIG180), or in other words, the completely blunt electrode. Each electrode geometry was used to weld one pass, forming one joint between two 100 mm × 50 mm plates.

### 2.2. Characterization Methodology

After welding, characterization of welds was done in order to assess the weld’s performance: macro-testing, microstructure examination on light microscope, scanning electron microscope, electron backscatter diffraction, and nanoindentation.

Macrostructural and microstructural examinations were performed on metallographically prepared specimens.

Cutting was done in the central part of the weld, 50 mm from the beginning of all welds (TIG60, ATIG30, ATIG60, ATIG90, ATIG180), which were macro-tested. Another set of metallographic specimens was cut 10 mm in the welding direction in selected welds (TIG60, ATIG60, ATIG90), which were used for microstructure examination on light microscope, scanning electron microscope, electron backscatter diffraction and nanoindentation. These measurements were used to assess spatial consistency within individual welds rather than statistical variation between repeated welds.

Metallographic cutting was performed using a Struers Discotom device (Struers, Ballerup, Denmark), with a grinding wheel and emulsion coolant. Cold mounting was used, with specimens placed in an aluminum mold and suspended in self-cured Arkema Veracryl polymethyl-methacrylate (PMMA) resin. After mounting, grinding was performed on the Struers Knuth Rotor device (Struers, Ballerup, Denmark), using a series of Silicon-Carbide abrasive papers, starting from grit P150, to P2000. After that, polishing was done on the Struers DP-U2 device (Struers, Ballerup, Denmark) with Buehler MetaDi 6, 3, 1 and ¼ µm diamond suspensions sprayed over the MicroCloth (Buehler, Leinfelden-Echterdingen, Germany) polishing cloth. Etching was performed using aqua regia (HNO_3_ + 3HCl). Prepared specimens were examined by Orthoplan light microscope -LM (Leitz, Wetzlar, Germany) and JSM-6460LV scanning electron microscope -SEM (JEOL, Tokyo, Japan) operating at 20 kV. Spot Energy-dispersive X-ray spectroscopy (EDS) analyses were performed by the Oxford Inca system. On the other hand, mapping EDS analysis was done on the Lyra 3 GMU Field Emission SEM (Tescan, Brno, Czech Republic), equipped with EDAX EDS system. Image processing was done by Fiji 2.9.0 software.

For the EBSD analysis, the specimens were prepared by sectioning, mounting, grinding, and polishing. The sectioned specimens were mounted in a graphite-based conductive mounting powder, ground using a P320 to P1200 grit set of Silicon-Carbide abrasive papers, and subsequently polished with 3 μm and 1 μm High-Tech (Allied, Cerritos, CA, USA) glycol-based polycrystalline diamond suspension on a TexMet C (Buehler, Leinfelden-Echterdingen, Germany) polishing cloth. A final polish was performed using 0.05 μm water-free colloidal silica suspension and Chem-Pol (Allied, Cerritos, CA, USA) polishing cloth. Grinding and polishing were carried out on an automated AutoMet 250 Pro Polisher/Grinder device (Buehler, Lake Bluff, IL, USA). Grain textures and structures were characterized using an EDAX EBSD system as part of a Lyra 3 GMU SEM (Tescan, Brno, Czech Republic) Field Emission SEM. The EBSD setup included an Octane Plus SDD detector and a Hikari High Speed Camera (EDAX, Mahwah, NJ, USA), with an accelerating voltage of 22 kV, beam intensity (BI) of 20, and step sizes of 0.3 μm for high and 1.5 μm for coarse resolution during the scanning process. Kikuchi pattern collection was performed at a 9 mm working distance with 6 × 6 binning. The acquired data was analyzed using the TSL OIM 8 (EDAX, Mahwah, NJ, USA) software, with each scan having 500,000 points and being cleaned by neighbor confidence index (CI) correlation, discarding all points with a CI less than 0.1.

Vickers microhardness was measured using the Tukon 1103 device (Wilson Hardness, Lake Bluff, IL, USA)., by applying a 100 g load (0.981 N) and a dwell time of 15 s. Measurements were done in four areas: central equiaxed part of the weld metal, columnar part of the weld metal, recrystallized area next to the melt line and base metal. In each of these areas, three indentations were made, with the average values reported, as well as standard deviations.

Nanoindentation was performed with at least 7 indents on each specimen using a iMicro Nano indenter (KLA Corporation, Milpitas, CA, USA) fitted with a Berkovich tip. The tests were depth-controlled, with an indentation depth of 1000 nm. The applied depth, indentation force, and contact stiffness are continuously recorded by the instrument. These measurements are then converted into elastic modulus and hardness values using the Oliver-Pharr indentation equations [25]. Load–displacement data analysis provided the contact penetration depth and the corresponding contact area, which were used to calculate the Young’s modulus and nanohardness of the material.

## 3. Results and Discussion

### 3.1. Macro Testing

**Figure 1:**
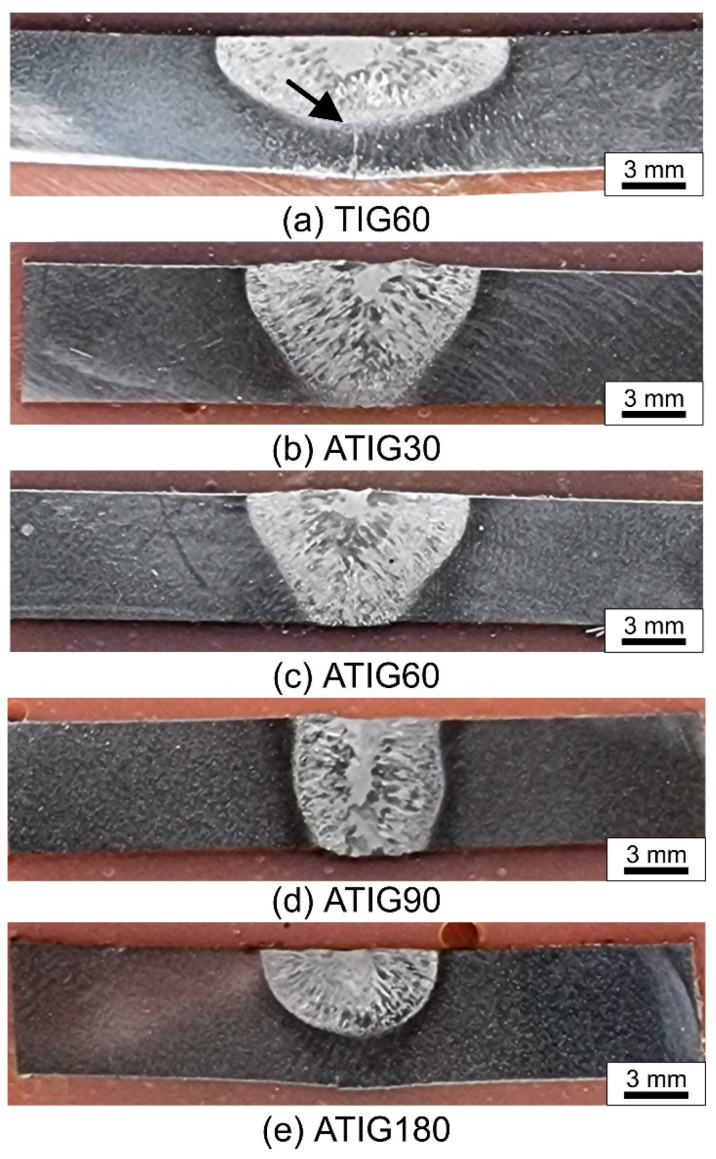
Macro-cross-sections (A) of welded specimens: (**a**) TIG 60; (**b**) ATIG 30; (**c**) ATIG 60; (**d**) ATIG 90; (**e**) ATIG 180.

Macro-images of the sectioned welded specimens are shown in Figure 1, cutting half of the welded line, 50 mm from the beginning of the plate. Weld dimensions: depth (D), width (W), D/W ratio and weld surface area (A, calculated using Fiji software) are presented in Table 2. It can be observed that compared to the TIG60 specimen, all specimens welded with the flux (ATIG30, 60, 90, 180) have a higher weld depth, which is the penetration, except for ATIG180. Also, all ATIG specimens exhibit a narrower weld face compared to the TIG60 specimen and, consequently, a higher depth/width ratio. The highest D/W ratio was obtained in the ATIG90 specimen, reaching a value close to 1.

Finally, it is clear that the effectiveness of the flux is coupled with the electrode tip geometry, which plays a major role in providing a larger weld surface area. The largest surface area was obtained in specimens ATIG30 and ATIG60, significantly more than in the control specimen TIG60. On the other hand, in specimens ATIG90 and ATIG180, a smaller weld surface area was obtained, in which ATIG180 does not penetrate fully through the specimen. This can be explained by the narrower arc during welding of the ATIG180 specimen, which influences the reduction of oxides on the surface, with an incomplete downward flow of molten metal, resulting in a reduced penetration.

Moreover, in specimens ATIG30, ATIG60 and ATIG90, no weld angular distortion was present, unlike TIG60 and ATIG180, where 7° and 5° were detected. This was caused by the crystallization of the relatively wide weld top side and the marked lack of complete penetration. Furthermore, this distortion caused the appearance of a crack in specimen TIG60, at the spot where two plates were joined (marked by an arrow).

It should be noted that specimen dimensions influence heat dissipation and cooling conditions during welding. Consequently, the absolute values of weld geometry and microstructural characteristics obtained in the present laboratory-scale specimens may differ from those observed in larger components. Nevertheless, the comparative trends reported here are considered reliable because all experiments were performed under identical specimen dimensions and welding conditions.

### 3.2. Microstructure and Microanalysis Testing

**Figure 2:**
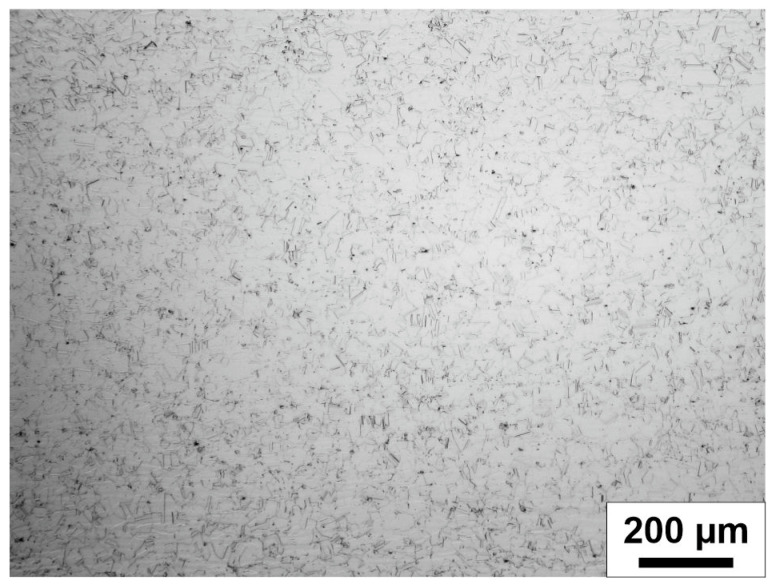
Microstructure of base metal.

**Figure 3:**
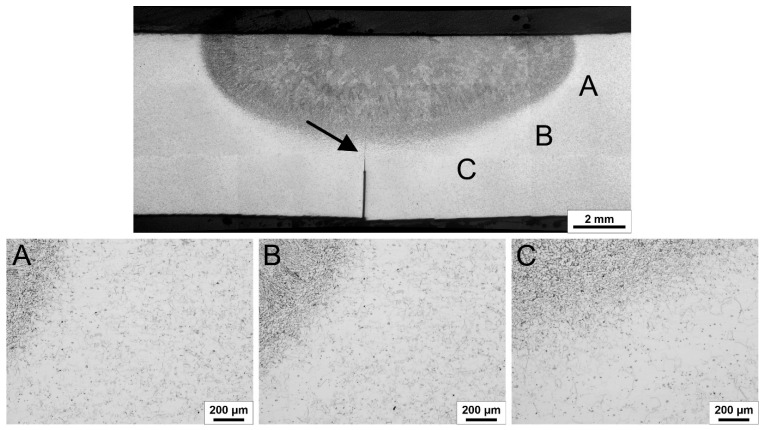
Macro-images of TIG60 with details depicting microstructure of recrystallized zones near the melt line, close to the top surface (**A**), center (**B**) and bottom (**C**).

**Figure 4:**
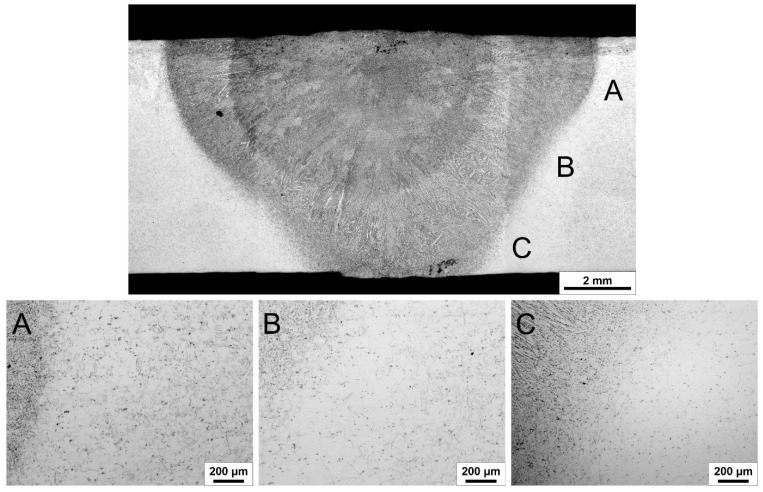
Macro-images of ATIG60 with details depicting microstructure of recrystallised zones near the melt line, close to the top surface (**A**), center (**B**) and bottom (**C**).

**Figure 5:**
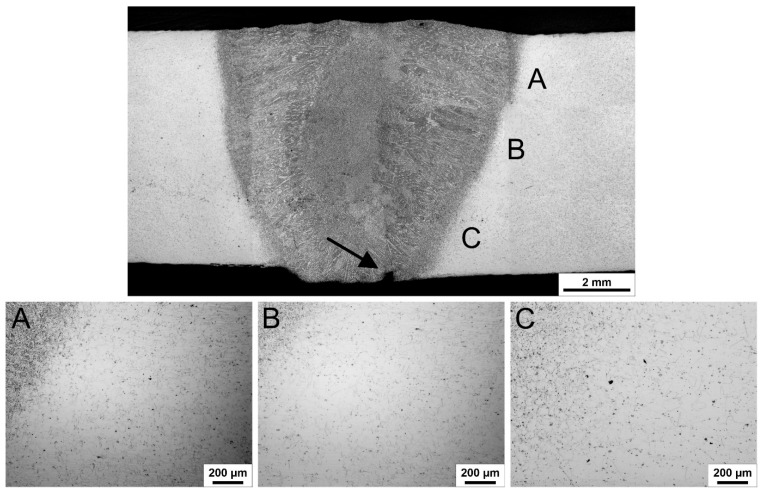
Macro-images of ATIG90 with details depicting microstructure of recrystallised zones near the melt line, close to the top surface (**A**), center (**B**) and bottom (**C**).

The microstructure of the base material is shown in Figure 2. It can be seen that the microstructure consists of polygonal austenitic grains, typical for IN-718. Specimens TIG60, ATIG60 and ATIG90 were further examined in more detail using a light microscope, on new specimens 10 mm away from the first sections in the welding direction (section B), Figure 3, Figure 4 and Figure 5. This was done in order to examine the selected specimens and compare them with the control specimen. The weld seam was evaluated in cross-sections taken at representative locations along the weld length. No defects were detected in the examined sections. While this suggests generally sound weld quality, a complete statistical analysis of the entire weld seam was not conducted, and therefore localized defects outside the examined regions cannot be fully excluded.

The crack present in specimen TIG60 is indicated by an arrow, as shown in Figure 3. Furthermore, the profile of welds ATIG60 (Figure 4) is similar to the one shown in Figure 1c. Specimen ATIG90 (Figure 5) has a slightly more pronounced V-shape compared to Figure 1d. In all specimens, a typical columnar weld microstructure was observed, with dendritic columns being oriented approximately 90° from the fusion line. These dendrites typically grow in the direction opposite to the heat flow, which is generally from the melt line toward the center of the weld pool. However, in the center of the weld, a more uniaxial microstructure was observed, formed later in the crystallization process. The dendrites are randomly oriented and equiaxed or exhibit a disordered morphology due to the interaction of opposing growth fronts [26,27].

Dimensions of the affected recrystallized areas near the melt line, measured from the melt line in parallel to the surface of the base metal, until reaching the unaffected base metal, are given in Table 3. The three values obtained are measured 1 mm from the top of the base metal surface, in the center section of the weld metal and in-line with the bottom of the weld metal. The illustration of the measurement sections is given within Table 3, shown in the example of specimen TIG60. It can be deduced that the narrowest distances D1 and D2 in the upper section of the weld in specimens ATIG60 and ATIG90 are lower than those in specimen TIG60. However, in the lower section, the narrowest is in specimen TIG60. Comparative weld depth, width, depth/weld ratio and weld surface area are given in Table 2, both for sections A and B. Although weld depth is the same in ATIG60 and ATIG90, achieving full penetration, weld width increases in these two specimens, depth to width ratio decreases, while weld surface area increases. On the other hand, in TIG60, penetration, weld width and surface area all decrease. That means the flux increases the melting potential of the arc compared to the non-flux specimen, where all weld dimension parameters decrease. On the other hand, data in Table 3 suggest that the width of HAZ in sections A and B (10 mm) away proves a high spatial consistency within individual welds.

In Figure 5, it can be observed that there is an irregularity in the weld root that is probably the consequence of a relatively narrow weld and slight misalignment of the welding process. For practical purposes, the ATIG60 specimen was considered to be worth being further tested and comparing to the control specimen TIG60.

**Figure 6:**
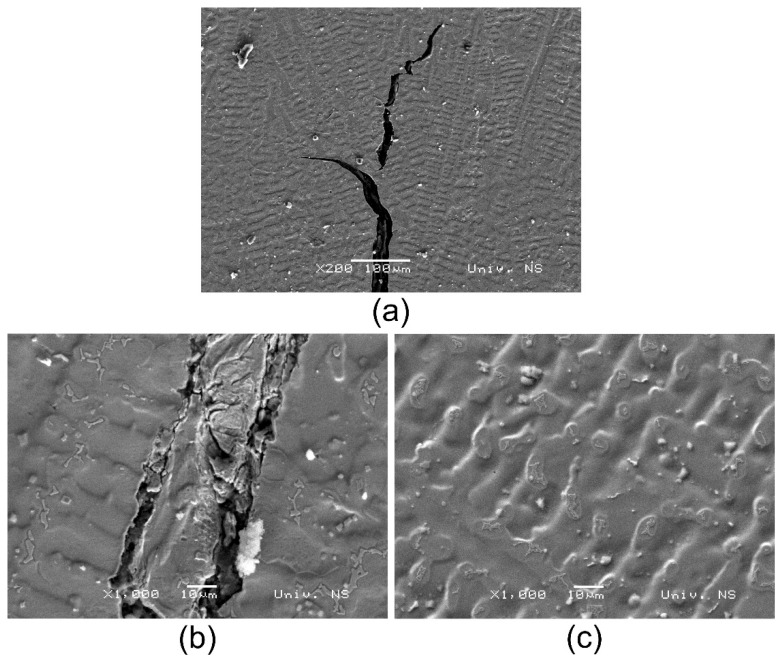
Microstructure of weld metal: (**a**) crack branching in weld metal—TIG60; (**b**) crack base with surrounding dendrites in specimen TIG60; (**c**) dendritic microstructure in specimen ATIG60.

Microstructures of specimens TIG60 and ATIG60 are shown in Figure 6. Weld metal microstructures are dendritic in both specimens, which is in agreement with the columnar macro-appearance shown in Figure 1 and Figure 2. The main difference is the existence of a crack that can be observed in specimen TIG60. This crack is shown in Figure 1a and is marked by an arrow. This is a hot crack (solidification crack) and forms during the solidification process of the weld metal, when the material is still at a high temperature. The interdendritic propagation means the crack is growing between the dendritic structures in the weld metal, during the cooling phase of the welding process, when residual stresses build up as the material solidifies. Cracks observed in the TIG60 condition indicate susceptibility to crack formation under the applied welding conditions. The present work does not attempt to quantify the statistical frequency of crack occurrence.

The morphology and interdendritic distance in specimens TIG60 and ATIG60 are 10.2 and 10.8 µm, respectively. This can be the result of a slower cooling speed of ATIG60 weld metal compared to TIG60 specimen [28]. Furthermore, a slower cooling speed in the ATIG60 specimen can be attributed to a shorter melt line of 16.8 mm, versus 19.2 mm in TIG60.

**Figure 7:**
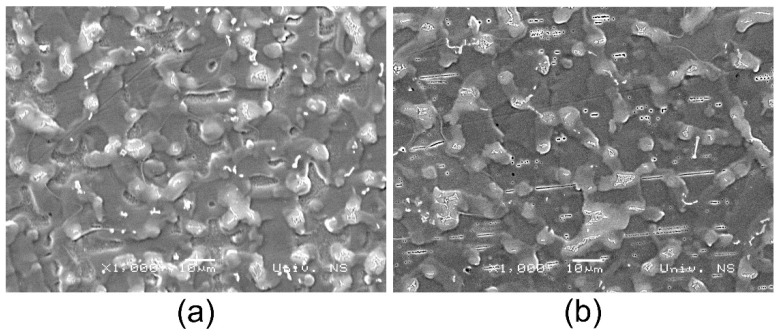
SEM images of the microstructure of dendrites in the central zone of the weld metal of: (**a**) TIG60; (**b**) ATIG60 specimen.

**Figure 8:**
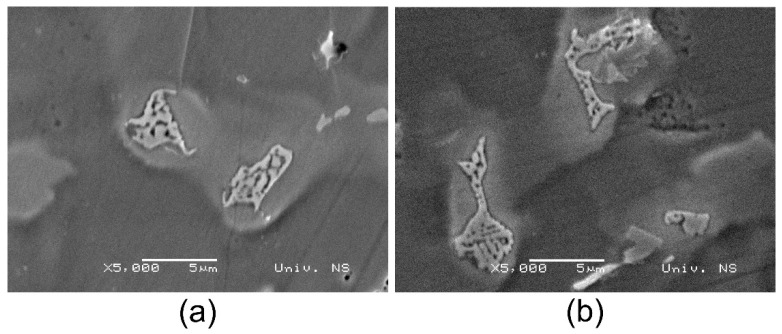
High-magnification SEM images of the microstructure of dendrites in the central zone of the weld metal: (**a**) TIG60; (**b**) ATIG60.

**Figure 9:**
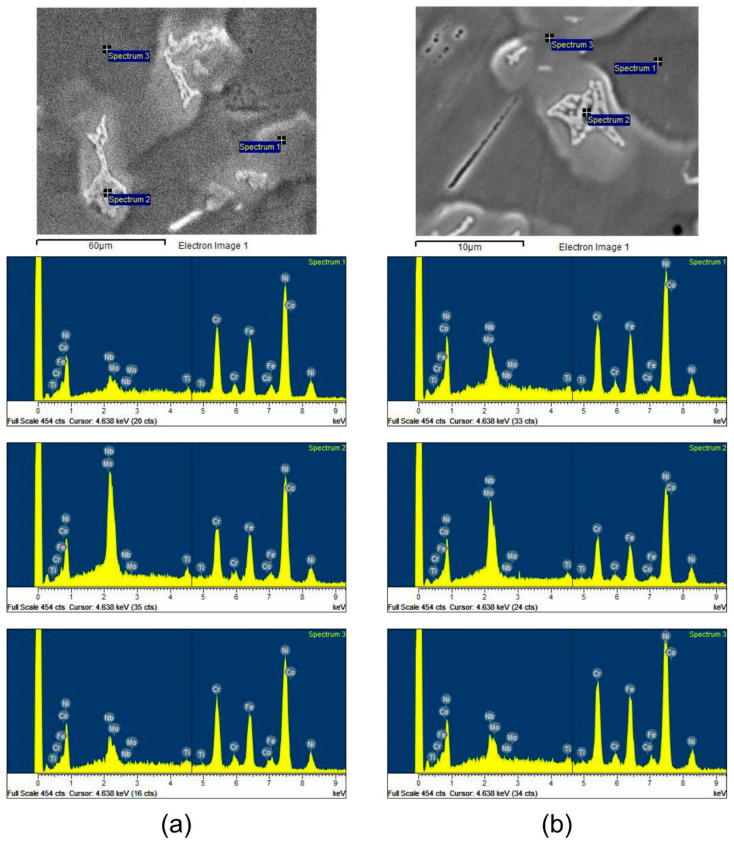
EDS analysis results of phases: (**a**) TIG60; (**b**) ATIG60.

High-resolution images of TIG60 and ATIG60 microstructures of the central weld-metal in the area of uniaxially oriented dendrites are shown in Figure 7 and Figure 8, while EDS analysis of present phases is given in Figure 9. It can be seen that there are two distinctive phases: the bright spider-web-like phase and the gray matrix. Gray matrix is a Ni-rich matrix with dissolved Fe, Cr, Co, Mo, and Ti, also called the γ-phase, which represents the bulk of the weld metal, and is usually tough and ductile. The bright phase can be identified as the Laves phase that forms after the formation of dendrites, that is, during the last solidification stage. It forms due to the segregation of certain elements, like Nb and Mo, in the non-equilibrium solidification conditions during welding. It is essentially an intermetallic phase. The presence of the whole range of elements in spectra is due to the influence of the surrounding material [29,30].

Energy-dispersive spectroscopy (EDS) of the identified phases in the ATIG weld metal did not reveal detectable levels of oxygen, nitrogen, or silicon originating from the sodium silicate-based activating flux. This observation is consistent with the widely accepted ATIG mechanism, where oxide fluxes primarily act through transient arc constriction and surface-active slag reactions rather than direct elemental transfer into the bulk weld metal. Previous studies on activated flux TIG welding of nickel- and iron-based alloys similarly report that the primary effect of oxide fluxes (like SiO_2_-, TiO_2_-, and Cr_2_O_3_-based systems) is to modify arc behavior and weld pool fluid flow, while significant incorporation of flux-derived elements into the weld metal is generally not observed [31,32].

The bright spider-web-like Laves phase is better depicted in Figure 8. It can be visually deduced that the Laves phases are larger and less coagulated in the TIG60 than in the ATIG60 specimen. Also, image processing revealed that the Laves phase is 4.622% of the whole surface area in TIG60 and only 2.746% in ATIG60 specimens. This, together with a more branched morphology and a larger size of individual Laves phases, means ATIG weld metal may be less prone to cracking, as the Laves phase is relatively hard and brittle [33]. A smaller, more coagulated Laves phase is usually an indicator of a faster cooling weld metal, where more Nb is trapped in the dendrite cores, reinforcing them. This is shown by the results in Table 4, where there is more Nb present in the dendrites in specimen ATIG60 compared to specimen TIG60. However, interdentritic distances and the length of the melt line in TIG60 suggest that this specimen cools more rapidly, which contrasts with what the Laves phase size and morphology in this specimen suggest. That means, a different mechanism is responsible for the coagulation and a smaller Laves phase size in ATIG60. This may be attributed to the increased weld pool stirring, that is, weld pool dynamics in the ATIG60 specimen, where enhanced weld pool convection promotes solute redistribution during solidification, reducing local enrichment of Laves-forming elements in interdendritic regions and thereby leading to a more refined and dispersed Laves phase morphology, which is in accordance with [34]. Therefore, the proposed mechanism should be considered qualitative and further detailed thermal and microstructural analysis is required to fully establish the relationship between weld pool dynamics and Laves phase formation.

### 3.3. EBSD Characterization Results

**Figure 10:**
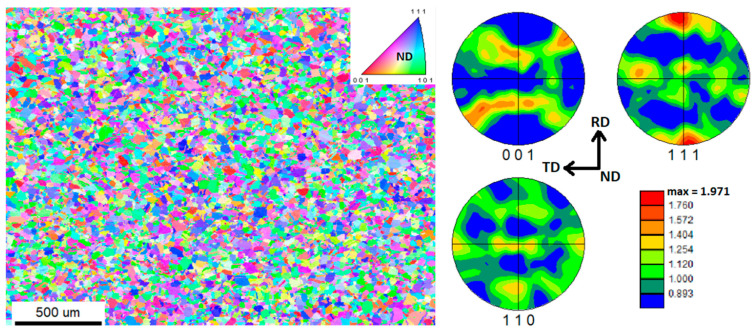
Inverse pole figure map and corresponding stereographic pole figure measured by EBSD for 718 initial material. Bottom-to-top direction on the maps aligns with the normal direction (ND). The IPF triangles provided in the corners of the maps describe the orientation of the crystal lattice frame with respect to the ND. ND is at the center of the pole figures.

Figure 10 presents an inverse pole figure (IPF) map and the corresponding pole figure of the initial structure of Inconel 718. IPF map reveals a relatively equiaxed grain structure with a pole figure showing relatively weak texture with a maximum intensity of 1.971.

**Figure 11:**
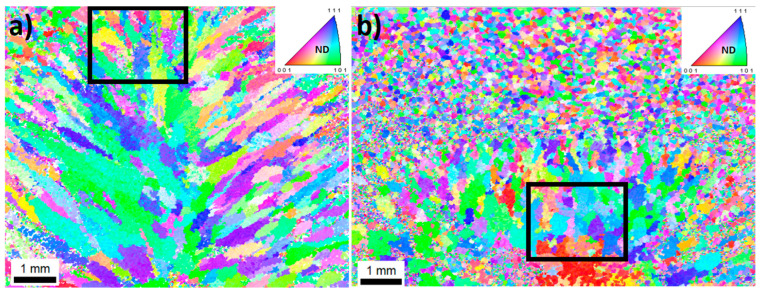
Low magnification IPF maps of: (**a**) ATIG60 and (**b**) TIG60 specimens. Bottom-to-top direction on the maps aligns with the normal direction (ND). The IPF triangles provided in the corners of the maps describe the orientation of the crystal lattice frame with respect to the ND. Black rectangles correspond to Figure 12.

Low-magnification IPF maps for ATIG60 and TIG60 specimens are presented in Figure 11. These maps correspond to the earlier SEM images in Figure 6, clearly demonstrating that the weld depth, and therefore the depth of microstructural changes, is significantly greater in the ATIG60 specimen compared to the TIG60 specimen. However, it is also evident that the weld width is larger in the TIG60 specimens when compared to the ATIG60 specimen, which is once again consistent with the images in Figure 6. Additionally, a significant grain growth was observed relative to the initial structure shown in Figure 10. In the case of ATIG60, the grain growth is more pronounced, with the grain aspect ratio being notably higher than that of the TIG60 specimens.

**Figure 12:**
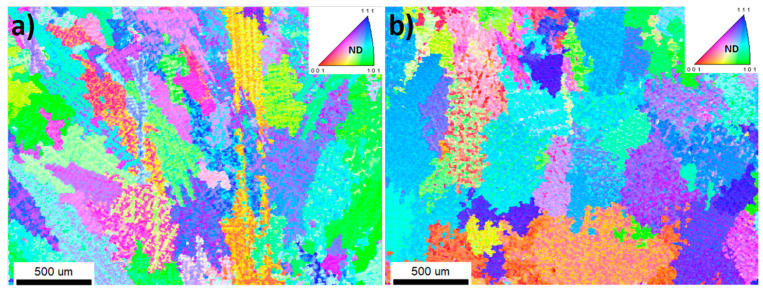
Corresponding IPF maps for the areas highlighted with rectangles in Figure 11: (**a**) ATIG60 and (**b**) TIG60 specimens, illustrating the dendritic structure within the grains. Bottom-to-top direction on the maps aligns with the normal direction (ND). The IPF triangles provided in the corners of the maps describe the orientation of the crystal lattice frame with respect to the ND.

Higher magnification IPF maps in Figure 12 validate some of the findings observed in Figure 10, confirming the presence of dendritic structures within the grains of the welds of both ATIG60 and TIG60 specimens.

### 3.4. Microhardness and Nanoindentation Testing

Microhardness values, measured in various areas: central equiaxed part of the weld metal (marked as A), columnar part of the weld metal (B), recrystallized area next to the melt line (C) and base metal (D), are shown in Table 5. These measurements were done at a distance of 1 mm from the base metal surface, towards the top of the weld. These areas closely correspond to Distance D1 presented in Table 3. It can be seen that in areas A, B and C, while in area D (base metal), similar values were obtained, which is the result of using the same base metal, that is, in the same rolled condition. Standard deviations are significantly lower in base metal (D) than in areas A to C. The higher microhardness of the matrix can be the result of a higher amount of Nb, as shown in Figure 9 and Table 4. Nb is the most significant alloying element that influences the strengthening of this alloy, through the formation of the Nb-rich Ni_3_Nb precipitates in IN-718 [30], which reflects on the microhardness, as in this case.

**Figure 13:**
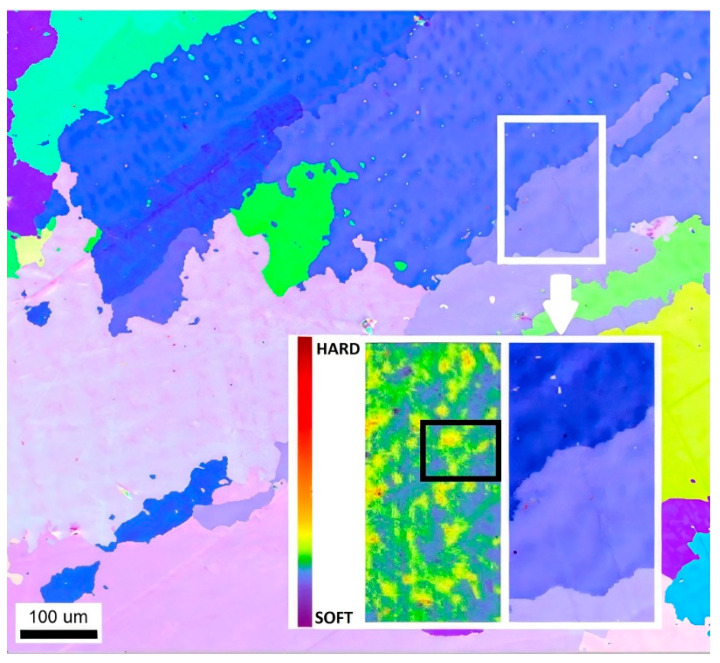
Hardness map and corresponding IPF map showing increased hardness of dendritic structure. Black rectangle corresponds to Figure 14.

**Figure 14:**
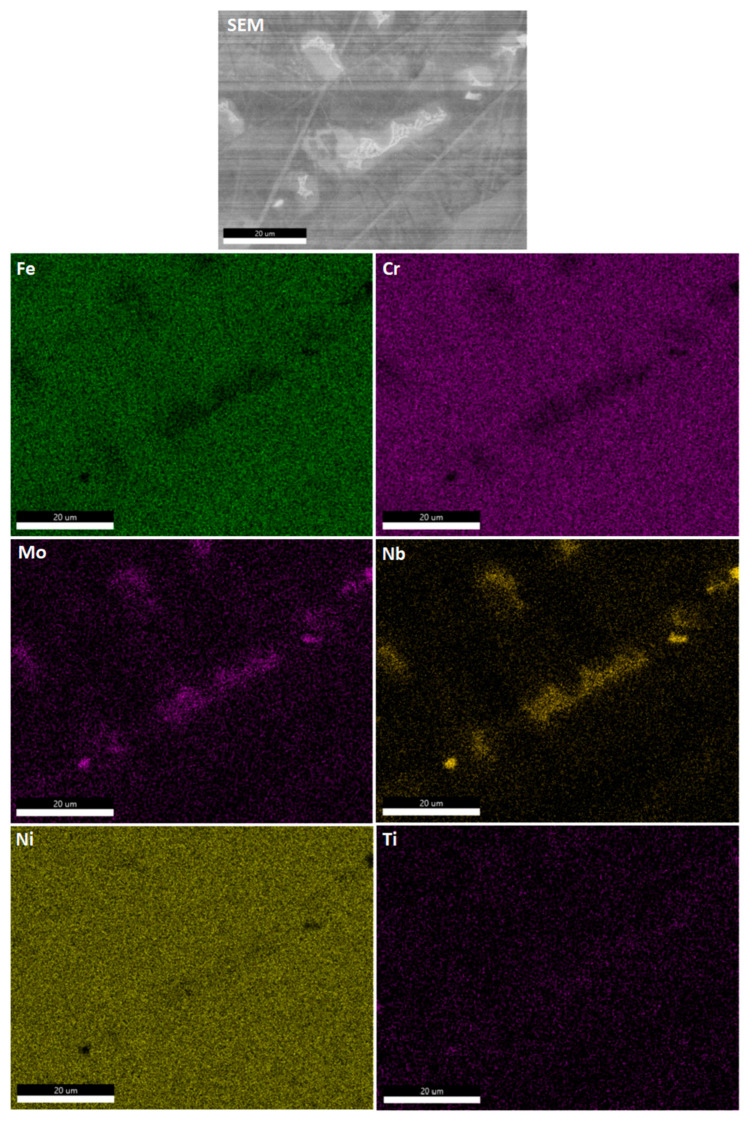
SEM image and corresponding EDS maps of Fe, Cr, Mo, Nb, Ni, and Ti for the area highlighted by the black rectangle in Figure 13.

Figure 13 depicts nanoindentation results in specimen ATIG60, while the black rectangle detail is shown at a higher magnification on a SEM micrograph and EDS analysis maps of selected chemical elements in Figure 14. It can be seen that the bright areas in Figure 14 show an elevated nanoindentation hardness compared to the matrix, which closely corresponds to the bright web-like area in the SEM image in Figure 14. EDS chemical analysis maps of the SEM area are shown in Figure 14. Elevated Nb and Mo content closely corresponds to the existence of the Laves phase. On the other hand, other element content, such as Fe, Cr and Ni, is significantly higher in the matrix, strengthening it, confirming the overall effect on its microhardness.

### 3.5. Molten Material Flow Model

**Figure 15:**
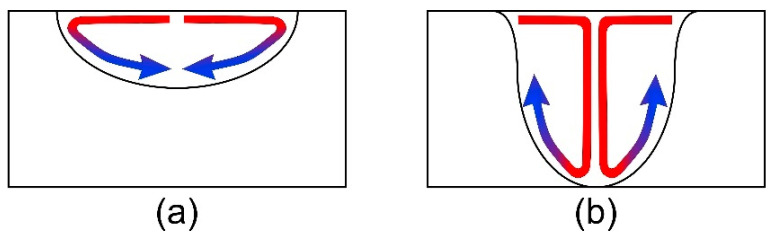
Molten material flow model (**a**) outward flow, shallow penetration. (**b**) inward flow, deep penetration - arrows indicate the direction of molten metal flow.

Based on the previous results, a molten metal material flow model can be devised, Figure 15. This model supports the hypothesis that the flux based on nanoparticles in sodium-silicate solvent that is not readily evaporable as alcohol and acetone [22] is effective in achieving the effect of reversal of the Marangoni convention, that is, the surface tension-driven flow. The interpretation of Marangoni convection reversal in ATIG60 is based on indirect evidence from weld morphology, microstructural modification in the form of the recrystallization zone near the melt line towards the base metal, as well as the microhardness measurements.

In the specimen welded without the novel flux (TIG60), the material flows from the low surface tension molten material in the centerline towards the edges, where the surface tension is higher, as explained in [10]. That drives the material from the centre of the molten surface towards the melting lines and then inwards into the material and back to the centreline, causing a relatively low penetration. In this process, the heat is dissipated mainly to the base metal on the sides, due to the relatively high temperature of the molten metal, transferring the highest heat towards this area. This results in a wider recrystallized area with coarser grains in the top area under the surface of the base metal. In this area, a lower microhardness was measured, as a clear indicator of larger recrystallized grains compared to the ATIG60 and ATIG90 specimens.

On the other hand, in specimens ATIG60 and ATIG90, the flux influences the reversal of high and low surface tension areas; there, the high surface tension is in the center, while the low surface tension is on the edges. Therefore, the molten metal flow is from the edges towards the center, and subsequently towards the base metal, melting and fusing the edges of the plates. Subsequently, the material flows along the melt line, towards the top surface of the base metal. That means the highest heat transfer is near the bottom of the base metal, resulting in a significantly wider recrystallized zone on the bottom, as shown in Table 3.

There are also differences between flux-welded specimens ATIG60 and ATIG90. In specimen ATIG 90, a higher D/W ratio is obtained (Table 2), with a narrower weld, but also a smaller weld surface area compared to ATIG60. Finally, in ATIG90, there is a defect in the root, which shows that the penetration was insufficient to completely melt the edge in the root, showing a somewhat lower fusion potential compared to the much wider ATIG60 that also has a more distinctive V-shape and a larger weld surface area. The main cause of a higher melting potential performed in ATIG60 is the more intensive downward flow, which is the result of a more concentrated arc, obtained by a lower angle of the electrode, but also, an initially wider weld might have influenced more space for the molten metal flow, a larger amount of molten metal, that forces a more intensive downward flow, also driven by inertial forces.

Weld pool dynamics is much more pronounced in the ATIG60 specimen, compared to the TIG60 specimen, since two hot molten material streams flow downwards towards the bottom of the weld, which is the main cause of a more intensive Nb incorporation into dendrites and a lower amount of the Laves phase. This mechanism proved to be dominant over the slightly lower cooling rate in ATIG60 versus TIG60, which has an opposite effect in a lower amount of Nb in dendrites.

## 4. Conclusions

Based on the presented results and within the aim and limitations of this study, the following conclusions can be drawn:

- Sodium silicate solvent proved to be effective in increasing the penetration of ATIG-welded IN-718 specimens. The highest effectiveness was achieved with the 30, 60 and 90o electrode tip angles, whereby no distortion occurred as in the TIG60 and ATIG180 specimens. Furthermore, in specimen TIG60, a crack was found in the weld metal as a result of distortion. Finally, specimens ATIG30 and ATIG60 had the highest weld surface area.
- A common columnar microstructure was obtained in all specimens, regardless of the presence of flux. In the central section of the weld metal, a uniaxial microstructure was obtained. Heat-affected zone width is larger near the weld surface and in the central section in the TIG-welded specimen, while in specimens welded with flux, it is higher at the bottom. This is due to the reversal of molten metal flow, as suggested by the proposed TIG and ATIG processing model.
- SEM/EDS analysis revealed Nb-rich interdendritic constituents consistent with the Laves phase formation. These constituents appeared less branched, smaller in size, and occupied a smaller area fraction in the ATIG60 specimen compared with TIG60. This observation suggests reduced Nb segregation and potentially greater Nb availability within the dendritic structure, which may contribute to the higher weld-metal microhardness observed in ATIG60. Nanoindentation measurements further indicated higher hardness values within the Nb-rich constituent regions than in the surrounding matrix.
- Sodium silicate combined with SiO_2_ and TiO_2_ nanoparticles described within the framework of US Provisional Patent Application # 63/362625 proved to offer significantly increased penetration in the ATIG process of IN-718. The novel flux produced weld geometries and HAZ distributions consistent with the Marangoni-flow reversal mechanism commonly reported for conventional ATIG fluxes. In addition, the sodium-silicate-based solvent offers improved storage stability and reduced volatility compared with conventional alcohol- and acetone-based carriers.

## Figures and Tables

**Table 1:** Chemical composition of IN-718 used as a base metal.

C	Cr	Fe	Mn	Mo	Ti	Al	Nb	Co	Ni
0.05	19.09	19.86	0.08	2.94	1.10	0.55	5.36	0.07	balance

**Table 2:** Weld dimensions and areas, sections A and B.

Specimen	Weld Depth D (mm)		Weld Width W (mm)		Depth/Width Ratio D/W		Weld Surface Area A (mm^2^)	
	A	B	A	B	A	B	A	B
TIG60	4.79	4.12	15.55	14.41	0.22	0.28	48.14	39.11
ATIG30	7	-	12.21	-	0.57	-	59.50	-
ATIG60	7	7	12.44	16.88	0.56	0.41	65.22	87.51
ATIG90	7	7	8.10	11.94	0.86	0.61	47.25	68.47
ATIG180	4.77	-	9.23	-	0.52	-	39.25	-

**Table 3:** Dimensions of the recrystallized areas form melt line to the unaffected base metal, sections A and B.

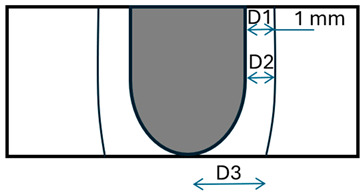	Width (mm)					
	TIG60		ATIG60		ATIG90	
	A	B	A	B	A	B
**Distance D1: 1 mm from the top of the base metal surface**	1.5	1.6	1.2	1.2	1.4	1.4
**Distance D2: Center section of the weld metal**	1.4	1.4	1.1	1.2	1.1	1.2
**Distance D3: In-line with the bottom of the weld metal**	2.7	2.8	4.8	5	3.3	3.4

**Table 4:** EDS quantitative results (wt.%).

Specimen	Ti	Cr	Fe	Co	Ni	Nb	Mo
**TIG60 Spectrum 1**	1.07	17.45	20.12	0.16	58.15	2.19	0.87
**TIG60 Spectrum 2**	1.82	13.82	14.68	0.96	50.37	15.33	3.02
**TIG60 Spectrum 3**	1.04	17.25	18.75	1.10	56.70	3.90	1.26
**ATIG60 Spectrum 1**	1.25	16.77	17.85	0.14	57.64	5.49	0.85
**ATIG60 Spectrum 2**	1.62	13.10	13.19	0.34	56.09	13.23	2.43
**ATIG60 Spectrum 3**	0.85	17.79	18.81	0.70	57.08	3.35	1.42

**Table 5:** Vickers microhardness average values and standard deviations measured in different areas.

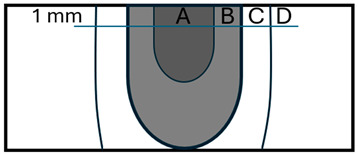		HV0.1			
		A	B	C	D
**Specimen**	**TIG60**	250.5 ± 7.2	247.9 ± 13.7	213.9 ± 10.4	232.6 ± 3.6
	**ATIG60**	269.3 ± 8.0	259.7 ± 14.9	231.8 ± 10.1	234.9 ± 4.4
	**ATIG90**	272.0 ± 8.2	258.3 ± 13.9	230.0 ± 9.2	235.1 ± 3.9

## Data Availability

The original contributions presented in this study are included in the article. Further inquiries can be directed to the corresponding author.
